# Human INO80/YY1 chromatin remodeling complex transcriptionally regulates the BRCA2- and CDKN1A-interacting protein (BCCIP) in cells

**DOI:** 10.1007/s13238-016-0306-1

**Published:** 2016-08-17

**Authors:** Jiaming Su, Yi Sui, Jian Ding, Fuqiang Li, Shuang Shen, Yang Yang, Zeming Lu, Fei Wang, Lingling Cao, Xiaoxia Liu, Jingji Jin, Yong Cai

**Affiliations:** 1 School of Life Sciences, Jilin University, 2699 Qianjin Street, Changchun, 130012 China; 2 Department of Gynecologic Oncology, The First Clinical Hospital of Jilin University, 71 Xinmin Street, Changchun, 130021 China; 3 National Engineering Laboratory for AIDS Vaccine, Jilin University, 2699 Qianjin Street, Changchun, 130012 China; 4 Key Laboratory for Molecular Enzymology and Engineering, The Ministry of Education, Jilin University, 2699 Qianjin Street, Changchun, 130012 China; 5 The first Hospital and Institute of Epigenetic Medicine, Jilin University, Changchun, 130061 China

**Keywords:** BCCIP, human INO80, chromatin remodeling complex

## Abstract

The BCCIP (BRCA2- and CDKN1A-interacting protein) is an important cofactor for BRCA2 in tumor suppression. Although the low expression of *BCCIP* is observed in multiple clinically diagnosed primary tumor tissues such as ovarian cancer, renal cell carcinoma and colorectal carcinoma, the mechanism of how BCCIP is regulated in cells is still unclear. The human INO80/YY1 chromatin remodeling complex composed of 15 subunits catalyzes ATP-dependent sliding of nucleosomes along DNA. Here, we first report that *BCCIP* is a novel target gene of the INO80/YY1 complex by presenting a series of experimental evidence. Gene expression studies combined with siRNA knockdown data locked candidate genes including BCCIP of the INO80/YY1 complex. Silencing or over-expressing the subunits of the INO80/YY1 complex regulates the expression level of BCCIP both in mRNA and proteins in cells. Also, the functions of INO80/YY1 complex in regulating the transactivation of BCCIP were confirmed by luciferase reporter assays. Chromatin immunoprecipitation (ChIP) experiments clarify the enrichment of INO80 and YY1 at +0.17 kb downstream of the *BCCIP* transcriptional start site. However, this enrichment is significantly inhibited by either knocking down INO80 or YY1, suggesting the existence of both INO80 and YY1 is required for recruiting the INO80/YY1 complex to *BCCIP* promoter region. Our findings strongly indicate that *BCCIP* is a potential target gene of the INO80/YY1 complex.

## Introduction

The BRCA2- and CDKN1A (Cip1, p21)-interacting protein BCCIP is alternatively spliced α and β isoforms (Liu et al. 2001; Ono et al. 2000). Both isoforms share N-terminal acidic domain (NAD, include 258 amino acids) and the internal conserved domain (ICD) (Liu et al. 2001). As an important cofactor for BRCA2 in tumor suppression, BCCIP has been implicated in many important cellular processes with obvious links to cancer. Knocking down BCCIP with siRNA in cells leads to defective DNA damage repair (Lu et al. 2005), abnormal cell cycle (Meng et al. 2004; Meng et al. 2004) and genomic instability (Meng et al. 2007). Recently, other colleagues and we have reported that *BCCIP* was down-regulated in several cancer tissues such as renal cell carcinoma, ovarian cancer and colorectal carcinoma (Meng et al. 2003; Liu et al. 2013). Thus, it is important to clarify the role of BCCIP in tumorigenesis, especially to know how the BCCIP is regulated in cells.

Yin Yang 1 (YY1), a member of the GLI-Krüppel class proteins, was first discovered as a DNA binding protein (Shi et al. 1991; Seto et al. 1991; Park and Atchison 1991; Hariharan et al. 1991). It is a ubiquitously expressed and evolutionarily conserved protein in human cells. Domain research found that YY1 includes not only an activation domain, but also contains a repression domain (Thomas and Seto 1999; Shi et al. 1997). In subsequent studies, YY1 has been clarified as a versatile protein which can either repress or activate gene transcription by recruiting different cofactors such as histone deacetylases (HDACs), methyltransferase enhancer of zeste homolog 2 (Ezh2), CREB-binding protein (CBP), and P300/CBP-associated factor (PCAF) (Yao et al. 2001; Lee et al. 1995).

Using biochemical purification approaches, we previously verified that YY1 is tightly associated with the human INO80 (INO80) chromatin remodeling complex (Jin et al. 2005). All evolutionarily conserved subunits (include actin-related proteins Arp4, Arp5, Arp8, Tip49a and Tip49b AAA^+^ ATPases, and hIes2 and hIes6) assembled on the conserved helicase-SANT-associated/post-HSA (HSA/PTH) and ATPase domains of INO80 protein (Jin et al. 2005). Both HSA/PTH and ATPase domains in INO80 protein are essential for catalyzing the ATP-dependent nucleosome remodeling activity of the INO80 complex. Based on YY1 with Arp4 and Arp8 together participate in assembling helicase-SANT-associated/post-HSA (HSA/PTH) module, suggesting that like other conserved subunits, YY1 is also essential for maintaining the ATP-dependent nucleosome remodeling activity of the INO80 complex (Chen et al. 2011). Experimental evidence indicates that *yeast* Ino80 is required for proper transcriptional regulation of many target genes (Morrison and Shen 2009), while in *Drosophila*, INO80 facilitates transcriptional repression of ecdysone-regulated genes during prepupal development (Neuman et al. 2014). Recent experimental results demonstrate that INO80 is also required in ESC self-renewal, somatic cell reprogramming, and blastocyst development (Wang et al. 2014). Although genome-wide studies show that INO80 binds many, but not all, loci in the genome (Moshkin et al. 2012), it is unclear whether there is a direct correlation between INO80 binding and target gene expression levels. One possibility is that YY1 as a transcription factor helps INO80 complex to recognize INO80-target genes and recruits the complex to some target genes such as CDC6 and GRP78 (Cai et al. 2007). In this study, using molecular and cell biology approaches, we first show that the BCCIP might be a novel target gene of the INO80 /YY1 chromatin remodeling complex. Our results will provide a strong theoretical basis to elucidate the BCCIP functions in cells.

## Results

### *BCCIP* was selected as a candidate target gene of the INO80/YY1 complex from gene expression profiles

**Figure 1:**
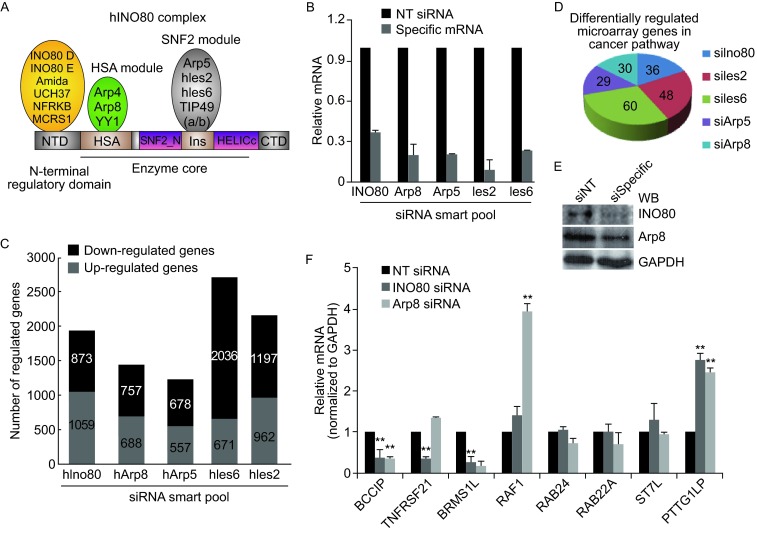
BCCIP is found in overlapping expressed gene profiles in INO80/YY1 complex-knockdown HeLa cells. (A) Functional domains of INO80/YY1 complex. NTD, N-terminal domain; HSA, helicase-SANT-associated domain; SNF2_N, SNF2 family N-terminal domain; Ins, insertion domain; HELICc, Helicase C-terminal domain; CTD, C-terminal domain. (B) Relative mRNA levels in siINO80, siArp8, siArp5, siIes2, and siIes6 knockdown HeLa cells. (C) Gene expression profiles in specific-siRNA knockdown Hela cells. Stacked column chart represents the total number of regulated genes in indicated specific siRNA knockdown HeLa cells. Each bar in the stacked column chart is composed of down- and up-regulated genes. (D) Differentially regulated microarray genes in cancer pathway in INO80/YY1 complex knockdown HeLa cells. (E) Knockdown efficiency. HeLa cells were transfected with 15 pmol INO80- or hArp8-siRNA and siNT (as control). (F) Verification of the mRNA of select genes from gene expression profiles. 48 h after siRNA transfection, RT-qPCR was performed to assess the relative mRNA levlel (*n* = 3). Bar graphs show ratios of RT-qPCR signals to GAPDH (all signals normalized to siNT). **P < 0.01 in comparison with siNT control (Student *t*-test)

Human INO80 complex is one of the most highly conserved chromatin remodelers. Eight core subunits (Arp5, Arp8, TIP49a/b, Ies2, Ies6, Arp4, and YY1) are evolutionary conserved and form an enzyme core including HSA and SNF2 modules. Except for conserved subunits, INO80 complex contains 6 metazoan-specific subunits which all assemble on N-terminus of INO80 protein and form an N-terminal regulatory module (Jin et al. 2005; Chen et al. 2011) (Fig. 1A). Increasing evidence suggests the functions of INO80/YY1 complex in gene transcriptional regulation (Morrison and Shen 2009; Conaway and Conaway 2009), but the precise mechanisms are still unclear. To investigate the target genes of the INO80/YY1 complex, total RNA from HeLa cells with specific siRNA (siINO80, siArp8, Arp5, siIes2 and siIes6) knocked down (Fig. 1B) were sent to EMTD Science and Technology Development Co., Ltd. (Beijing, China) for DNA microarray. As shown in Fig. 1C, a total of 1932, 1445, 1235, 2707 and 2159 genes were differentially expressed among INO80, Arp8, Arp5, hIes6 or hIes2 and NT siRNA knockdown HeLa cells, respectively. Hundreds of overlapping genes are found to be regulated by INO80, Arp8, Arp5, hIes6 and hIes2, which are components of the HSA and SNF2 module. On the other hand, a total of 602 genes were co-regulated by INO80 and Arp8 which participate in assembling HSA module (data not shown). Selected overlapping co-regulated genes (8 down and 6 up) in INO80/YY1 complex knockdown HeLa cells are shown in Table 1. mRNA from INO80- and Arp8- siRNA knockdown cells (Fig. 1E) was measured with RT-qPCR (Fig. 1F). Compared to NT siRNA control, selected gene mRNA including BCCIP, TNFRSF21, and BRMS1L was down-regulated with siRNA knockdown of INO80 and Arp8 in HeLa cells. In contrast, RAF1 and PTTG1IP mRNAs was up-regulated. However, there was no change of mRNA of RAB24, RAB22A, and ST7L. Table 2 compared the results of microarray illumina and RT-qPCR. In addition, differentially expressed genes (DEGs) in INO80/YY1 complex knocked down cells were used for KEGG annotation by using DAVID web annotation tool. DEG-enriched pathways in INO80 complex knockdown cells were shown previously (Chen et al. 2011). Fig. 1D shows the differentially regulated microarray genes in cancer pathway after knocking down the indicated subunits of INO80/YY1 complex.

**Table 1:** Select overlapping co-regulated genes in INO80/YY1 knockdown gene expression profiles

Gene symbol	Gene ID	Gene description	Up or Down	Fold change
AMHR2	NM_020547	Anti-mullerian hormone receptor, type II	Down	−3.58
BCCIP	NM_016567	BRCA2 and CDKN1A interacting protein	Down	−3.62
CDH1	NM_0043609	Cadherin 1, type 1	Down	−4.97
CCL28	NM_019846	Chemokine (C-C motif) ligand 28	Down	−2.61
FGFBP1	NM_005130	Fibroblast growth factor binding protein1	Down	−3.65
GRTP1	NM_024719	Growth hormone regulated TBC protein 1	Down	−3.21
TPD52	NM_001025252	Tumor protein D52	Down	−2.35
WTIP	NM_059037	Wilms tumor1 interacting protein	Down	−3.15
CDKN1A (p21)	NM_078467	Cyclin-dependent kinase inhibitor 1A	Up	6.92
EGFR	NM_005228	Epidermal growth factor receptor	Up	9.5
GAS1	NM_002048	Growth arrest-specifc 1	Up	4.0
MBD1	NM_015845	Methy-CpG binding domain protein 1	Up	5.26
RAF1	NM_002880	v-raf1 murine leukemia viral oncogene homolog1	Up	4.41
TGFBR1	NM_004612	Transforming growth factor, beta receptor 1	Up	4.56

Genes shown in table are the selected overlapping expressed genes in at least three subunits of INO80/YY1 complex knockdown HeLa cells

**Table 2:** Comparison of the results of the microarray-illumina and qPCR

Gene symbol	Gene ID	siINO80		siArp8	
		Illumina	qPCR	Illumina	qPCR
BCCIP	NM_016567	Down	Down	Down	Down
TNFRSF21	NM_014452	Down	Down	Down	–
BRMS1L	NM_032352	Down	Down	Down	Down
RAF1	NM_002880	Up	–	Up	Up
RAB24	NM_001031677	Up	–	Up	–
RAB22A	NM_020673	Down	–	Down	–
ST7L	NM_138729	Up	–	Up	–
PTTF1IP	NM_004339	Up	Up	Up	Up

Co-regulated genes selected from different specific subunit of the INO80/YY1 complex knockdown gene expression profiles are tested by quantitative PCR (qPCR). The performed qPCR results were then compared with DNA microarray illumine data

### The expression of BCCIP was regulated by INO80/YY1 complex in HeLa and 293T cells

**Figure 2:**
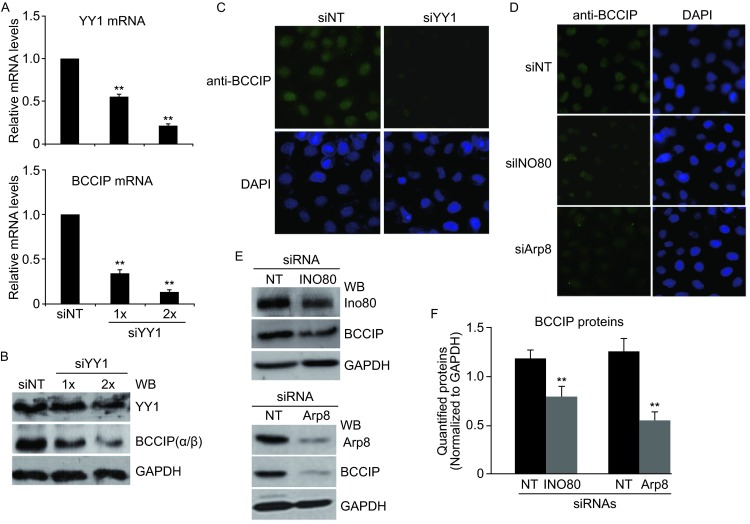
The INO80/YY1 chromatin remodeling complex is implicated in regulating BCCIP expression in HeLa cells. (A) Down regulation of BCCIP mRNA in siYY1 knockdown HeLa cells. Relative mRNA levels of YY1 and BCCIP were measured by RT-qPCR. Each bar represents the mean of 3 independent experiments. **P < 0.01 in comparison with siNT-transfected control (Student *t*-test). (B) Reduction of BCCIP protein levels by knocking-down YY1 in HeLa cells. Whole cell lysate from siYY1 knockdown cells was analyzed by western blot. Specific proteins were detected by indicated antibodies (GAPDH as internal control). Anti-BCCIP antibody recognizes both α- and β- isoform of BCCIP. (C and D) Immunofluorescence stained BCCIP proteins. HeLa cells were transfected with YY1-, INO80-, and Arp8 siRNA. 48 h later, immunofluorescence staining was carried out using BCCIP antibody. DAPI staining shows total nuclei. (E) Western blot analysis of BCCIP proteins in INO80 (upper) and Arp8 (down) knocked down HeLa cells. Prepared whole cell lysate from INO80- or Arp8- siRNA knocked down HeLa cells were analyzed by western blot. Quantified protein levels were shown in (F). **p < 0.01 in comparison with siNT-transfected control (Student *t*-test)

UCSC (The University of California-Santa Cruz) Genome Browser at http://genome.ucsc.edu provides timely, convenient access to a database of high-quality genome sequence and annotation (Karolchik et al. 2013). Based on UCSC database (ChIP-Seq) search, we found that YY1 was restricted to the *BCCIP* transcriptional start site proximal region in different types of cell lines including human lung carcinoma type II epithelium-like A549, human liver hepatocellular carcinoma HepG2 and human colon cancer HCT116. To clarify this observation, we first examined the BCCIP expression level in YY1-siRNA knockdown HeLa cells. As shown in Fig. 2A, dose-dependent down-regulation of *BCCIP* (lower panel) in YY1-siRNA knockdown (upper panel) HeLa cells was confirmed by qPCR. Furthermore, low protein levels of BCCIP in YY1-siRNA knockdown HeLa cells were also detected by western blot (Fig. 2B) and immunofluorescence staining (Fig. 2C) approaches, respectively. Given that YY1 is a subunit of the INO80 chromatin remodeling complex, the implication of INO80 complex in regulating BCCIP is speculated. To determine whether BCCIP is a potential target gene of the INO80/YY1 complex, we evaluated the BCCIP expression level in INO80- and Arp8- (evolutionary conserved subunit of the INO80 complex) siRNA knockdown HeLa cells. Declined protein levels of BCCIP by knocking down INO80 and Arp8 in HeLa cells were presented by western blot (Fig. 2E and 2F) and immunofluorescence staining (Fig. 2D), suggesting the involvement of INO80/YY1 complex in regulating the BCCIP expression.

### Human INO80 facilitates the transactivation of BCCIP

**Figure 3:**
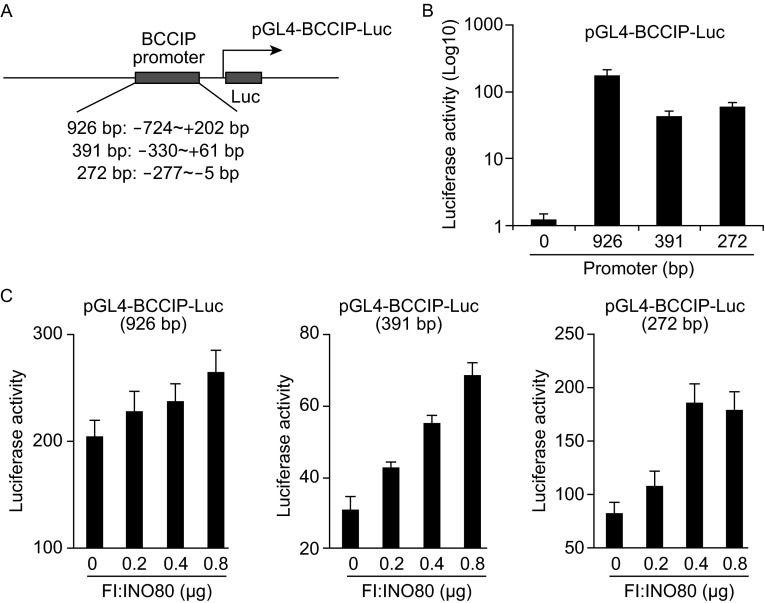
Transactivation of BCCIP is facilitated by INO80/YY1 chromatin remodeling complex. (A) Schematic of construction of pGL4-BCCIP-Luc plasmids. A length of 926 bp (−724 ~+202 bp), 391 bp (−330 ~+61 bp), and 272 bp (−277 ~ −5 bp) region of BCCIP promoter was introduced into pGL4-Luc vector. The luciferase activity of each plasmid was shown in B. (C) A dual luciferase assay. pGL4-BCCIP-Luc vectors and INO80wt plasmid (0.2, 0.4 and 0.8 μg) were co-transfected into 293T cells. 48 h later, luciferase activity in the lysates was determined by a dual luciferase assay. Error bars represent data from three independent experiments

To further explore whether the INO80/YY1 chromatin remodeling complex affects the transactivation of BCCIP, three designed BCCIP promoter region was sub-cloned into pGL4 luciferase vector (Fig. 3A). Constructed pGL4-BCCIP-Luc plasmids containing different BCCIP promoter region were first tested in dual luciferase assay (Fig. 3B). Then, the impact of INO80 on BCCIP transactivation was estimated by co-transfection of pGL4-BCCIP-Luc with Flag-INO80 plasmids. As shown in Fig. 3C, in contrast to a basal-level luciferase activity, co-transfection of pGL4-BCCIP-Luc and INO80 dose-dependently increased BCCIP-luciferase activity in all cases, indicating the roles of INO80 complex in regulating the transactivation of BCCIP.

### Chromatin remodeling activity of the INO80 complex might be associated with regulation of BCCIP expression

**Figure 4:**
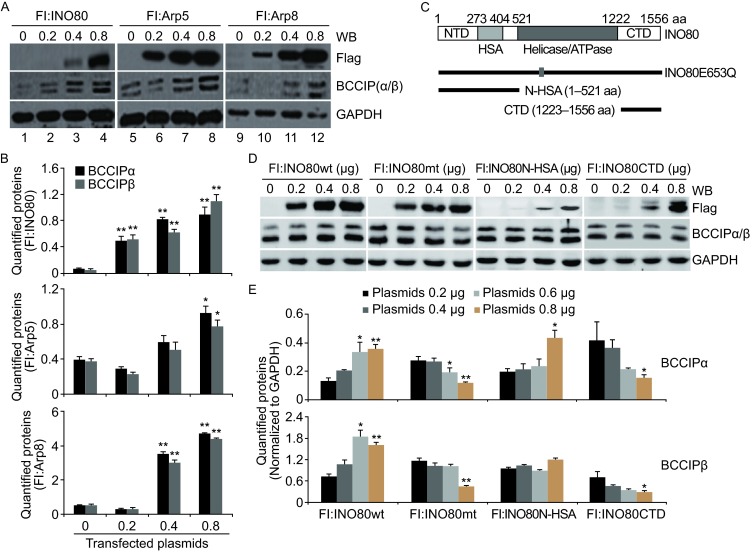
Chromatin remodeling activity of the INO80/YY1 complex might be important for regulating the BCCIP expression. (A) Facilitation of BCCIP expression by INO80/YY1 complex. 293T cells were co-transfected with pGL4-BCCIP (contains 391 bp promoter region) -Luc vector and Flag tagged INO80, Arp5, and Arp8 pcDNA plasmids (0.2, 0.4 and 0.8 μg). 48 h later, Cells were harvested and whole cell lysate was prepared. BCCIP proteins were checked by western blot with anti-BCCIP antibody which can recognize both BCCIPα and BCCIPβ. Here shows representative figures of western blot analysis. (B) Quantified proteins. Western blot images from (A) experiments were quantified with densitometry using Quantity One Basic software (BioRad). Bar graph shows the standard error of the mean of three independent experiments. *P < 0.05, *P < 0.01 in comparison with pcDNA3.1-vector transfected control (Student *t*-test). (C) Point or delete mutation of INO80. (D) Effect of INO80 protein on BCCIP expression. pGL4-BCCIP-Luc (contains 391 bp promoter region)vector was co-transfected with Flag tagged INO80wt, INO80mt (E653Q), INO80 N-HSA, and INO80CTD pcDNA plasmids (0.2, 0.4 and 0.8 μg) into 293T cells. Prepared whole cell lysate was subjected to western blot analysis. (E) Quantified proteins. Western blot images from (D) three independent experiments were scanned and quantified with densitometry using Quantity One Basic software (BioRad). *P < 0.05, *P < 0.01 in comparison with pcDNA3.1-vector transfected control (Student *t*-test)

To investigate the effects of INO80 complex on BCCIP-mediated transactivation, pGL4-BCCIP-Luc vector containing 391 bp promoter region (−330 ~+61 bp) was used in subsequent experiments. As shown in Fig. 4A, co-transfection of pGL4-BCCIP-Luc vector with Flag-tagged INO80, Arp5, and Arp8 increased not only luciferase activities (data not shown) but also intracellular BCCIP proteins in a dose-dependent manner. Compared to pcDNA3.1 control group, statistically significant differences were found in INO80 (*P* < 0.01 at 0.2, 0.4, and 0.8 μg of plasmids, respectively), Arp5 (*P* < 0.05 at 0.8 μg of plasmids), or Arp8 (*P* < 0.01 at 0.4 and 0.8 μg of plasmids, respectively) transfected 293T cells (Fig. 4B), suggesting the importance of enzyme core including HSA and SNF2 modules of the INO80 complex in regulating BCCIP expression. To further confirm this result, point or delete mutations of INO80 protein were designed (Fig. 4C). BCCIP protein levels in overexpressed INO80wt, INO80mt, INO80 N-HSA, and INO80CTD proteins in 293T cells were analyzed by western blot with anti-BCCIP antibody. Consistent with previous results, increased tendency of both BCCIPα and BCCIPβ was detected in INO80wt over-expressed 293T cells (Fig. 4D, left panel). However, this phenomenon is reversed by over-expression of INO80mt (E653Q, a DEAD/H box point mutation) which interfere with INO80 ATPase activity. As a result, BCCIP proteins are gradually decreased by increasing the transfection amount of INO80mt (Fig. 4D, second panel from the left), demonstrating the importance of ATPase activity of the INO80 complex in BCCIP expression. In addition, except for BCCIPα at 0.8 μg transfection of INO80 N-HSA (*P* < 0.05), little changes of BCCIP proteins were observed in INO80 N-HSA transfected 293T cells (Fig. 4D, third panel from the left). Furthermore, co-transfection of pGL4-BCCIP-Luc vector with INO80CTD, decreasing tendency of both BCCIPα and BCCIPβ was represented in a dose dependent manner, and the statistically significant differences were obtained at 0.8 μg transfection of INO80CTD (Fig. 4D, right panel). Above experimental results indicate that enzymatic activities of the INO80 complex might be associated with the regulation of BCCIP expression. Quantified proteins of BCCIPα and BCCIPβ for Fig. 4D are shown in Fig. 4E.

### INO80/YY1 chromatin remodeling complex is restricted to the BCCIP transcriptional start site proximal region

**Figure 5:**
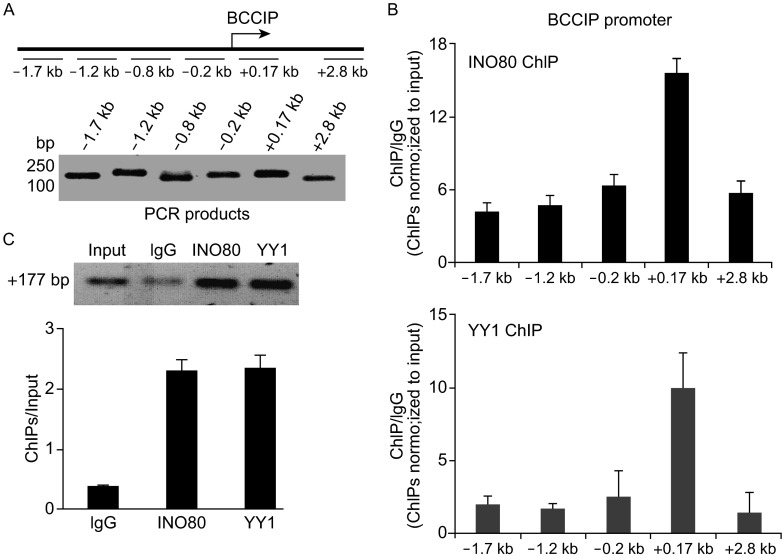
INO80 co-localize with YY1 at the BCCIP promoter. (A) Seven primer sets designed for amplifying ChIP DNA (upper panel). PCR products from each primer set were confirmed using 2.5% agarose gel (lower panel). (B) Co-occupying of INO80 and YY1 at the *BCCIP* promoter region. ChIP assays were performed using INO80 (upper panel) or YY1 (lower panel) antibodies. ChIP DNA was analyzed by qPCR. Bar graph shows the ratios of ChIP DNA signals (normalized to input) to IgG (also normalized to input). Error bars represent the standard error of the mean of three independent experiments. (C) Enrichment of INO80/YY1 at the +0.17 kb downstream of the BCCIP transcriptional start site. qPCR product at +0.17 kp downstream of BCCIP transcriptional start site was visualized by ethidium bromide (upper panel) in 2.5% agarose gel. Quantified PCR signals (Quantity One software) were analyzed by *t*-test (lower panel). Error bars indicate mean ± SE, and the significant difference is expressed as **P < 0.01 (Student *t*-test)

**Figure 6:**
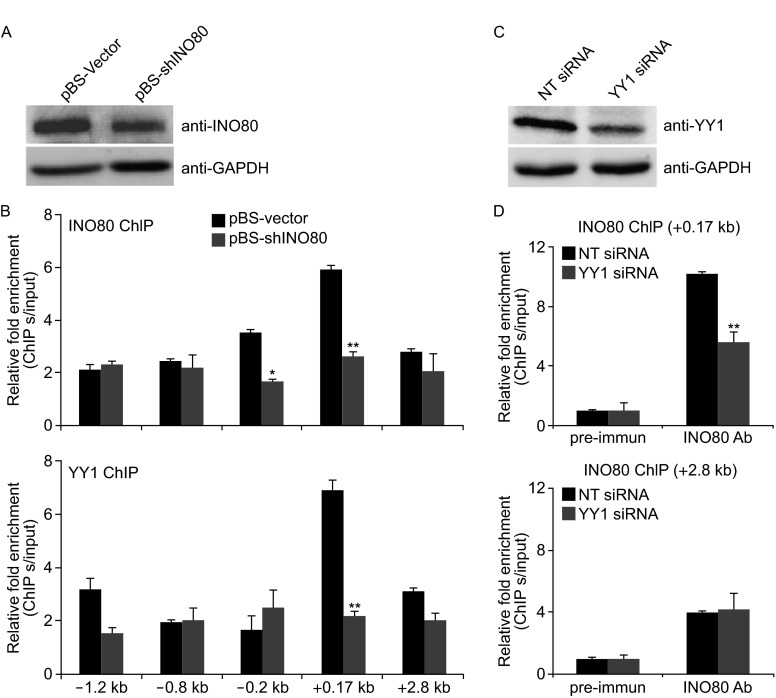
The INO80 chromatin remodeling complex may activate BCCIP gene through YY1. INO80- and YY1- ChIP in siINO80 knockdown 293T cells were performed. INO80 knockdown efficiency was shown as A. ChIP DNA was analyzed by qPCR (B). Y-axis shows the ratio of ChIP DNA signals to IgG. All ChIPs were normalized to input. Error bars represent mean ± SE (*n* = 3). * *P* < 0.05, ** *P* < 0.01 in comparison with pBS empty vector control (Student *t*-test). (C) Confirmation of YY1 knockdown. (D) INO80 ChIP in YY1-knockdown 293T cells. INO80 ChIP was carried out in YY1 knocked down 293T cells. +0.17 kb and +2.8 kb promoter primer sets were used in ChIP experiments

Data in the previous experiment clearly show that BCCIP level in both mRNA and protein, and the transactivation of BCCIP in cells is regulated by INO80/YY1 chromatin remodeling complex. To further determine whether the *BCCIP* is transcriptionally controlled by INO80/YY1 complex, indicated primer sets were designed to amplify ChIP DNA (Fig. 5A, upper panel). Amplified ChIP DNA by each specific primer set for BCCIP promoter proximal region as well as the region far away from the transcriptional start site was confirmed by qPCR, and visualized in 2.5% DNA agarose with Ethidium bromide (Fig. 5A, lower panel). Human INO80 and YY1 specific antibodies were then used in ChIP assays. As shown in Fig. 5B, both INO80 (upper) and YY1 (lower) are recruited at +0.17 kb downstream of the *BCCIP* transcriptional start site, indicating the co-localization of INO80 and YY1 on the *BCCIP* promoter region. Further analysis of performed qPCR products on 2.5% DNA agarose gel acquired a significant enrichment both in INO80 and YY1 ChIP at +0.17 kb downstream of the *BCCIP* transcriptional start site (Fig. 5C). However, knockdown INO80 by transfecting pBS-shINO80 (Fig. 6A) in 293T cells significantly blocked the recruitment of INO80 and YY1 at the +0.17 kb downstream of the *BCCIP* transcriptional start site (*P* < 0.01 in both cases), confirming the binding of INO80/YY1 complex at *BCCIP* promoter region (Fig. 6B). Similarly, silencing YY1 by siRNA (Fig. 6C) in 293T cells inhibited the recruitment of INO80 at the +0.17 kb downstream of the *BCCIP* transcriptional start site (*P* < 0.01), but not at +2.8 bp far away from the downstream of the *BCCIP* transcriptional start site, suggesting the requirement of YY1 for binding of INO80 to *BCCIP* promoter region (Fig. 6D).

## Discussion

It has been known that the INO80 complex is highly conserved from yeast to human. Evolutionarily conserved subunits including YY1 form an enzyme core which is composed of two modules (HSA and SNF2) assembled on the conserved HSA/PTH and ATPase domains of the INO80 protein (Fig. 1A). Although the precise functions of those two modules remain to be explored, actin-related protein Arp4 and Arp8 have been reported to bind to DNA and histones, suggesting the contributions of the INO80 complex in recognition of DNA and/or nucleosome substrates (Seeber et al. 2013; Shen et al. 2003). On the other hand, recognition of DNA and/or histones by subunits of INO80 complex is beneficial to the ATP-dependent nucleosome remodeling activity, thereby regulating gene expression through chromatin structure alteration. Based on the analysis of performed gene expression profiles from INO80-, Arp8-, Arp5-, hIes6- and hIes2-knockdown HeLa cells, we not only clarified that over thousands of genes were regulated in each gene expression profile, but also found that hundreds of genes were co-regulated by silencing the subunits which composed of SNF2 (Arp5, Ies6 and Ies2) and HSA (Arp8) modules (Cao et al. 2015). Also, enrichment of differentially expressed genes in multi-KEGG pathways including pathways in cancer was confirmed by KEGG pathway enrichment analysis, indicating the importance of the INO80 complex in cellular biological processes such as cancer pathway (Fig. 1D) (Chen et al. 2011).

We previously reported that zinc-finger transcription factor YY1 is tightly associated with human INO80 chromatin remodeling complex, and with actin-related proteins (Arp4 and Arp8) together, YY1 assembles HSA module on the HSA/PTH domain of INO80 protein, demonstrating the roles of YY1 in maintaining the nucleosome remodeling activity of the INO80 complex (Chen et al. 2011; Chen et al. 2013). More importantly, as an essential co-activator, YY1 can recruit the INO80 complex to some target genes such as CDC6 and GRP78 (Cai et al. 2007). Based on our gene expression profiles and screening experiments, *BCCIP* was chosen as one of the potential candidates of target genes of the INO80 complex. Based on database search in UCSC Genome Browser, we found that YY1 is restricted at the BCCIP transcriptional start site proximal region in different types of cancer cell lines including A549, HepG2 and HCT116, suggesting the functions of YY1 or INO80/YY1 complex in regulating the expression of *BCCIP*.

In this report, we first verified that *BCCIP* is a novel target gene of the INO80 chromatin remodeling complex by presenting a series of experimental evidence. The levels of BCCIP both in mRNA and protein were not only regulated by silencing- or over-expressing- YY1 (Fig. 2), but also were changed by knocking down INO80 and Arp8 or over-expression of INO80, Arp5, and Arp8 HeLa cells (Figs. 2 and 4A), suggesting the roles of INO80/YY1 complex, but not limited to YY1, in regulating the BCCIP expression. ChIP assays further verified that INO80 and YY1 co-localize the *BCCIP* promoter region, and the binding site to BCCIP gene was enriched at +0.17 kb downstream of the transcriptional start site (Fig. 5). However, this enrichment was significantly inhibited in either INO80- or YY1- knocking down 293T cells (Fig. 6), suggesting that (i) INO80/YY1 complex binds to the *BCCIP* promoter region; (ii) binding of YY1 to the *BCCIP* promoter region requires INO80; (iii) INO80 complex is required for YY1 to gain access to target gene promoters and to activate transcription of target genes.

In summary, we first demonstrate that BCCIP might be a novel target gene of the human INO80/YY1 chromatin remodeling complex. This result will provide a theoretical basis for further understanding and elucidating the functions of BCCIP in tumorigenesis and cellular processes.

## MATERIALS AND METHODS

### Antibodies

Anti-BCCIP (16043-1-AP) antibody was obtained from Proteintech Group (China, Wuhan). Anti-YY1 (sc-1703X) antibody, rabbit IgG (sc-2027) and mouse IgG (sc-2025) were purchased from Santa Cruz Biotechnology (U.S.A.). Anti-Flag (M2)-Agarose and monoclonal antibody (F3165) were from Sigma (U.S.A.). Anti-INO80 (NM_017553, residue 1–526 aa), anti-Arp8 (NM_022899, full length), and anti-GAPDH (NM_002046, full length) rabbit polyclonal antibodies were raised against bacterially expressed proteins (Jilin University).

### Cell culture/maintenance

HEK293T (human embryonic kidney) and HeLa (human cervical cancer) cells were grown on plates at 37 °C in DMEM (Dulbecco’s modified Eagle’s Medium, GIBCO, Life Technologies^TM^, U.S.A.) with 10% fetal bovine serum (KangYuan Biology, China) and 1% Penicillin-Streptomycin mixture (Thermo Fisher Scientific, U.S.A) in the presence of 5% CO_2_.

### Reverse transcription PCR (RT-PCR)

Total RNA was isolated using RNAiso Plus (Takara, Japan) and reverse transcribed to cDNA using PrimeScript 1st Strand cDNA Synthesis Kit (Takara, Japan). The resulting cDNA was analyzed by quantitative real time PCR (qPCR) with Eco^TM^ Real-Time PCR System (Illumina, Gene Company Limited) (China, Hongkong). All PCR products were synthesized using the following program: initial denaturation step was conducted at 95 °C for 30 s, followed by 35 cycles of denaturation at 95 °C for 5 s, annealing at 60 °C for 30 s and extension at 72 °C for 30 s. The following primer sets were used for qPCR analysis: YY1, 5′-CCCTCATAAAGGCTGCACAA-3′ (forward) and 5′-TGAACCAGTTGGTGTCGTTT-3′ (reverse), yielded a 147-bp product; Arp8, 5′-CCAGGCTGAGAAGGGTGATA-3′ (forward) and 5′-GCAGGAAGAGTGTCTGTGGC-3′ (reverse), yielded a 200-bp product; BCCIP, 5′-TCAAGAGTTGGTTCTACGCTTC-3′ (forward) and 5′- CATGGGCAGAGCGATCTGT-3′ (reverse), yielded a 111-bp product; INO80, 5′-CGGAATCGGCTTTTGCTA-3′ (forward) and 5′-TGTCGGCTGGTCAGTTGG-3′ (reverse), yielded a 292-bp product; and the internal control GAPDH, 5′-ATCACTGCCACCCAGAAGAC-3′ (forward) and 5′- ATGAGGTCCACCACCCTGTT-3′ (reverse), yielded a 443-bp product.

### Transient transfection

cDNAs encoding the INO80 full length (NM_017553), INO80 point mutation (E653Q), deletion mutations including INO80 N-HSA (residue 1–521 aa), INO80CTD (residue 1223–1556 aa), Arp8 (NM_022899), Arp5 (BC038402), and YY1 (BC065366) proteins were sub-cloned with Flag tags into pcDNA3.1(-). 293T cells cultured in 6-well tissue culture plates were transiently transfected with Flag-tagged plasmids (1–2 μg) using PEI (Cat. 23966, Polysciences, U.S.A.) according to the manufacturer’s recommendation. The ratio of plasmids (μg) and PEI (μL) is 1:2. 48 h later, the whole cell lysate was prepared and the proteins were isolated using SDS-PAGE gel (10%). The specific proteins were detected by western blot with specific antibodies.

### siRNA/shRNA knockdown

HeLa cells were transfected with 10–20 pmol INO80- (D-004176), YY1- (sc-36863), Arp8- (Customized) and non-targeting (NT)- siRNAs (D-001206, as control) for knocking down specific genes. INO80-, Arp8- and non-targeting (NT)- siRNAs were ordered from Dharmacon (U.S.A.). YY1 siRNAs were purchased from Santa Cruz Biotechnology (U.S.A.). 48 h after transfection, total RNA or whole-cell extract was prepared for RT-PCR and western blot analysis. In ChIP assay, 293T cells were transfected with pBS-shINO80 plasmid (10 μg/10 cm culture plate) or YY1 siRNAs for knocking down INO80 and YY1.

### Luciferase reporter assay

BCCIP promoter region (926 bp, −724 ~+202 bp; 391 bp, −330 ~+61 bp; 272 bp, −277 ~ −5 bp) was introduced into pGL4-Luc vector. Similar to previously described (Wu et al. 2013), 293T cells grown on 12-well plates were co-transfected with 0.4 μg of pGL4 which encodes firefly luciferase, 0.12 ng of the control plasmid Renilla luciferase vector which encodes renilla luciferase and effector plasmid expressing pGL4-BCCIP using PEI reagent (Polysciences, U.S.A.). Total effector plasmids in each transfection were adjusted to 0.8 μg with empty vector. 48 h after transfection, the transactivation activity of pGL4-BCCIP was determined by measuring firefly and *Renilla* luciferase activities using the Dual-Luciferase Reporter assay kit (Promega, U.S.A.).

### Immunofluorescence staining

~30% confluence HeLa cells in 24-well plates containing a coverslips (8D1007, Nest) on each well were transfected with INO80-, Arp8- and YY1- siRNAs using RNAiMAX (Invitrogen). After 48 h, cells were permeabilized with 0.3% TritonX-100 and incubated with BCCIP antibody (1:200) at room temperature, then stained with FITC-conjugated secondary antibody (rabbit/green: 1:300, sc-2012). Cell nuclei were stained with DAPI containing Vectashield (Vector Laboraries, Inc. H-1200). Fluorescence images were observed with Olympus BX40F Microscope (Olympus Corporation).

### Chromatin immunoprecipitation (ChIP) assays

1 × 10^7^ of 293T cells grown to 80% of confluence were used for each ChIP. INO80 and YY1 antibodies were used in ChIP assay. ChIP’d DNA was subjected to qPCR. Each experiment was performed 2–3 independent times. Both specific antibody- and IgG-ChIP signal were normalized to total input. The following primer sets were used in qPCR: BCCIP −1.7 kb (−1769 ~ −1603 bp), 5′-GACAGCTTCCTCTCTTCTCCA-3′ (forward) and 5′-ATTTCGGTCTTTCCTGGGAGT-3′ (reverse); −1.2 kb (−1281 ~ −1093 bp), 5′-CCCTCCACTCTTCTCTCCAAA-3′ (forward) and 5′-CCCTCCACTCTTCTCTCCAAA-3′ (reverse); −0.8 kb (−860 ~ −694 bp), 5′-CTCGAACTCCTGACCTCTGGTG-3′ (forward) and 5′-CTGGAGCGCATTTCTTGACC-3′ (reverse); −0.2 kb (−205 ~ −53 bp), 5′-AGCTTTGGGAGGAGTGATGGAG-3′ (forward) and 5′-TAGCCAAGGGGGTGAGGAAC-3′ (reverse); +0.17 kb (+177 ~+353 bp), 5′-TCATTGACGAGGTGAGAAGGAC-3′ (forward) and 5′-GCAGTGTGTTAAAGACCGAGGAG-3′ (reverse); +2.8 kb (+2841 ~+2984 bp), 5′-GCTGCCGTGTCTGGTTTATTTG-3′ (forward) and 5′-GCCAAGATTGCCATCTGTGAAG-3′ (reverse).

### DNA microarray

Specific genes (include hINO80, Arp5, Arp8, Ies2 and Ies6) knocked down with siRNAs in HeLa cells and the total RNA sent to EMTD Science and Technology Development Co., Ltd. (Beijing, China) for DNA microarray analysis. The Illumina microarray datasets were accessible from the National Center for Biotechnology Information Gene Expression Omnibus (GEO) data repository (http://www.ncbi.nlm.nih.gov/geo/) using the series accession number GSE68655. The online biological classification tool DAVID (Database for Annotation, Visualization and Integrated Discovery) was used to perform the enrichment analysis (Huang da et al. 2009; Huang da et al. 2009), Gene Ontology (GO) (Ashburner et al. 2000) function analysis and Kyoto Encyclopedia of Genes and Genomes (KEGG) (Ogata et al. 1999) pathway enrichment analysis of differentially expressed genes (DEGs). During the analysis, *P* < 0.05 and FDR < 0.02 genes used in the annotation were defined as statistically significant.

### Statistics

Two-tailed student’s *t*-test was used in this study. Data shown is mean + SD from at least three independent experiments. Statistical probability is expressed as *P < 0.05, **P < 0.01.

## Acknowledgments

This work was supported by the National Natural Science Foundation of China (Grant Nos. 31071131 and 31171245), by the National Laboratory of Biomacromolecules (O5SY02110A and 2012kf04), and by the Project of Jilin Province Science and Technology Development Program (20130413002GH).

## Abbreviations

BCCIP, BRCA2- and CDKN1A (Cip1, p21)-interacting protein; HSA/PTH, helicase-SANT-associated/post-HSA; ChIP, chromatin immunoprecipitation; qPCR, quantitative real time PCR; DEGs, differentially expressed genes; GO, Gene Ontology; KEGG, Kyoto Encyclopedia of Genes and Genomes; GEO, Gene Expression Omnibus.

## Compliance with ethical standards

Jiaming Su, Yi Sui, Jian Ding, Fuqiang Li, Shuang Shen, Yang Yang, Zeming Lu, Fei Wang, Lingling Cao, Xiaoxia Liu, Jingji Jin and Yong Cai declare that they have no conflict of interest. This article does not contain any studies with human or animal subjects performed by any of the authors.

## Author contributions

JS, YS, JD, FL, SS, YY, ZL and LC performed experiments. JJ and FW analyzed data. XL provides reagents. JS, YC and JJ designed experiments and drafted the manuscript. All authors read and approved the final manuscript.
